# Intensive Outpatient Treatment of Depression in a Spinocerebellar Ataxia Type 1 Patient

**DOI:** 10.1155/2019/9186797

**Published:** 2019-02-11

**Authors:** Eric Black

**Affiliations:** Assistant Professor of Psychiatry, Southern Illinois University, USA

## Abstract

**Objective:**

Spinocerebellar ataxia type 1 (SCA1) is but one subtype of spinocerebellar ataxia (SCA), each of which can possibly be considered a separate neurological condition (N. Whaley, S. Fujioka, Z. K. Wszolek, 2011). SCA is hereditary, progressive, and degenerative. SCA1 symptoms initially include coordination problems and ataxia. SCA1 can also include speech and swallowing difficulties, spasticity, ophthalmoplegia, cognitive difficulties, and even sensory neuropathy, dystonia, atrophy, and fasciculations. Literature has established that depressive symptoms can be exhibited with spinocerebellar ataxia patients regardless of type (T. Schmitz-Hübsch, 2011). While a higher risk for depression occurs with more severe SCA disease, successful treatment to mitigate symptoms has been documented (N. Okamoto, M. Ogawa, Y. Murata, et al., 2010). In this case a SCA1 patient with advanced neurological disease was enrolled in a psychiatric intensive outpatient (IOP) treatment program in the midwestern United States to address his comorbid depressive symptoms. This treatment option allowed a less restrictive environment while providing a more structured therapeutic setting and social support for the patient, much more so than that which is typically offered in a traditional outpatient setting.

**Case Report:**

A patient with relatively advanced SCA1 successfully participated in a psychiatric IOP program or depressive symptoms and benefitted from the program's structure and additional psychosocial support.

**Conclusion:**

Awareness among physicians, particularly psychiatrists and neurologists, regarding IOP programs as a treatment option for comorbid depression in the clinical setting of progressive SCA or other neurological conditions can be beneficial to patients requiring an increased level of psychiatric treatment.

## 1. Introduction

Patients with various subtypes of SCA can exhibit depressive symptoms. The pathology of such depressive symptoms in the setting of SCA is not fully explained [1, 2]. Either the symptoms clinically arise due to stress resulting from progressively decreased neurological motor function, or there possibly exists a neurodegenerative process manifesting in depression. The treatment of major depressive disorder (MDD) complicated by SCA has been reported in the literature [2–4]. The benefits of pharmacotherapy, specifically utilizing selective serotonin reuptake inhibitors (SSRIs) which have proven effective even when depression occurs with advanced SCA, points to reversible abnormalities of serotonin transmission as a depression etiology [3]. This successful treatment, however, does not completely exclude the possibility that the depressive symptoms were, at least in part, reactive to the stress from decreased motor function in SCA. Numerous SCA analyses have been done, producing data ranging from basic quantitative SCA survival data [5], to patient-reported outcome measures (PROMs) as a complement to neurological scales [6]. PROMs including depressive symptoms within the Patient Health Questionnaire (PHQ-9) have been tracked, underlying the importance of PROMs as additional outcome measures for SCA patients [6]. To this end, the specific management and treatment of depression arising from the patient's progressive SCA functional limitations need even further attention in the literature. This unique aspect of depression treatment is addressed in this report, describing a SCA1 patient with advanced neurological disease enrolled in a psychiatric IOP program to provide social support in a structured therapeutic setting. The utilization and effectiveness of this type of psychosocial intervention for depression in the setting of SCA is not mentioned in the literature as part of a treatment plan for a patient. However, IOPs in general have been utilized successfully for a variety of psychiatric conditions [7]. The additional longitudinal emotional and psychological support along with monitored pharmacotherapy with SSRIs improved the quality of life for a SCA1 patient despite the progression of his neurological symptoms. The objective is to highlight that this more comprehensive treatment option, when available to SCA patients suffering from depression, may warrant more consideration from neurologists and psychiatrists in the management of this chronic condition.

## 2. Case Report

A 49-year-old Caucasian male was evaluated at the Department of Adult Psychiatry Intensive Outpatient (IOP) Treatment Program for initial intake after having been referred from the Department of Neurology Outpatient Clinic. He had complaints of depressed mood, lack of enjoyment of pleasurable activities, sleep disturbances, poor concentration, and occasional passive suicidal thoughts. These symptoms were in association with ongoing progression of symptoms related to his diagnosis of SCA1. His neurologic symptoms included coordination problems and ataxia initially at diagnosis, but in recent years speech impairment, swallowing difficulties, some spasticity, and eventually muscle atrophy were presented. He had to utilize a motorized wheelchair as a result of his progressive disability. His current house was not fully wheelchair accessible. He lived alone but had siblings who visited at least several times per week. He also seemed to lack insight into the relationship between his thoughts, feelings, and physical limitations.

At the psychiatric evaluation, he expressed that his self-esteem was strongly affected by his limited physical mobility due to his SCA1, and he seemed extremely unhappy. He reported passive death wishes at least once a week, usually without a suicidal plan or intent. Laboratory evaluations including hemogram, liver function tests, total protein, vitamin B12, folic acid, T3, T4, and TSH were within normal limits. Baseline psychiatric evaluation with the Beck Depression Inventory (BDI-II) [8] revealed scores of 23 (moderate depression). According to clinical evaluation as well as DSM-V [9] criteria, the patient was diagnosed with major depressive disorder and he was started on sertraline 50 milligrams (mg) by mouth per day. He had never taken medications for depression before. Group cognitive behavioral therapy focusing on negative cognitions as well as aspects of dialectical behavioral therapy were initiated as part of the IOP protocol [10]. These group sessions occurred three times a week for several hours a session totaling approximately 12 hours per week. Individual cognitive behavioral therapy with a psychologist also occurred weekly for the first several weeks of the IOP program. He also met with a psychiatrist weekly, then every two weeks, and finally monthly for medication management. His sertraline was increased over this time period to 100 mg per day. He reported compliance with medications and reported no side effects. He also participated in 3 family sessions whereby his family members were also engaged in psychoeducation and therapy. Partial response to treatment was observed at the 12th week with reduction of BDI-II [8] score to 12 (mild mood disturbance). He participated in the IOP program for approximately 6 months in total, becoming increasingly more engaged, and eventually reported a subjective significant reduction in depressed mood, anhedonia, and suicidal thoughts. His sleep and concentration also improved. He reported improved insight into the mind-body connection between his physical and emotional conditions. He acquired new coping skills to limit counterproductive behaviors and irrational emotional responses to his occasional negative thoughts. With help of IOP staff and social workers, he found an assisted living accepting of his limited ambulation, moving to a better housing situation. He then was eventually scheduled for medication management and monitoring follow-up appointments in the Department of Adult Psychiatry Outpatient clinic once discharged from the IOP treatment program.

## 3. Limitations

While this case report fills a niche in the literature regarding the role of an IOP in treating comorbid depression in the setting of a chronically deteriorating condition such as SCA1, there are some limitations involved. Firstly, there are numerous varieties of SCA, and this case focuses on a patient with SCA1. It may serve as an area of future research when other depressive symptoms are studied in other subtypes of SCA. Also, pharmacotherapy (an SSRI) was used in this case to treat MDD, and this modality is not exclusive to an IOP program's offerings. Reversible abnormalities of serotonin transmission as a depression etiology in SCA patients have been noted [3], and this aspect of treatment can be addressed in any psychiatric setting, not just an IOP. PROMs in past research have included PHQ-9 as a measure of depression in SCA patients [6], and here the BDI-II [8] was utilized as an outcome measure. While both are valid instruments in clinical practice, future research may lead to more standardization in this specific patient population. The length of time the patient participating in the IOP program was about 6 months, which can vary among different countries and regions. The availability, strength of services offered, and the experience of the IOP program staff compared to traditional clinics can all vary as well. This unfortunately may be the greatest limitation, speaking to the need for more promotion and development of such valuable psychosocial programs for patients.

## 4. Discussion

In clinical psychiatric practice, treating patients with comorbid physical impairments can be challenging as the scope of their overall impairment may be better addressed through a broader variety of approaches [11, 12]. Standard psychiatric outpatient medication management clinics are typically challenged for resources. They may lack extensive social and psychological services that patients with physical disabilities need to more globally assess their overall condition as it affects their mental health, representing a significant disparity for those patients [12]. IOP treatment programs, particularly those in the United States where this case occurred, offer unique resources and longitudinal structure providing intensive, goal-oriented, group-based programming to foster increased self-awareness, and behavioral change. Target areas for intervention in a robust IOP program with a full array of social workers and psychologists include symptom management, self-esteem and emotional regulation, interpersonal effectiveness, distress tolerance, and adaptive coping [7]. Addressing substance use has traditionally been part of United States IOP programs as well [13]. Programs also include routine monitoring of the patients by a psychiatrist. As was noted earlier, such an extensive IOP program unfortunately may not always be available, pointing to a glaring need in certain mental health communities.

In the present case, although response was achieved through psychological and pharmacological treatment with respect to the patient's MDD, the report of success through an IOP treatment program specifically in a comorbid SCA1 patient is notable. This favorable outcome points to a possibly better alternative than a traditional psychiatric outpatient clinic for patients with significant psychosocial stress due to progressive physical limitations. It should be noted that the level of care provided in traditional psychiatric clinics and services offered can vary greatly, so some may compare more favorably to IOP programs. This case also illustrates that the IOP program availability and promotion in the community must be robust, and here the initial referral came from a Neurology outpatient clinic as staff there were aware of the program, believing it to be a good fit for the patient's needs. It is noted again here too that such resources may not be available in all countries or regions.

In conclusion, physicians, especially psychiatrists and neurologists, should remain mindful of the utility of IOP programs as treatment options for mental health conditions. The more intensive psychosocial treatment can be particularly beneficial for those patients with chronic physical conditions, especially those with significant psychiatric comorbidities, such as SCA. Pharmacological targets for therapy and the stress of living with a chronic progressive physical disease are optimally addressed in such a program. Healthcare professionals owe this holistic approach to their patients as part of a comprehensive model of caring for them.
